# Microfibers in the Diet of a Highly Aerial Bird, the Common Swift *Apus apus*

**DOI:** 10.3390/toxics12060408

**Published:** 2024-06-03

**Authors:** Alessandra Costanzo, Roberto Ambrosini, Milo Manica, Daniela Casola, Carlo Polidori, Valentina Gianotti, Eleonora Conterosito, Maddalena Roncoli, Marco Parolini, Beatrice De Felice

**Affiliations:** 1 Department of Environmental Science and Policy, University of Milan, Via Celoria 26, I-20133 Milan, Italy; roberto.ambrosini@unimi.it (R.A.); carlo.polidori@unimi.it (C.P.); marco.parolini@unimi.it (M.P.); beatrice.defelice@unimi.it (B.D.F.); 2 Gruppo Insubrico di Ornitologia OdV, Via Manzoni 21, Clivio, I-21050 Varese, Italy; milomanica@gmail.com (M.M.); danycasola@gmail.com (D.C.); 3 Dipartimento dello Sviluppo Sostenibile e della Transizione Ecologica, Università del Piemonte Orientale, Piazza S. Eusebio 5, I-13100 Vercelli, Italy; valentina.gianotti@uniupo.it (V.G.); eleonora.conterosito@uniupo.it (E.C.); 20025041@studenti.uniupo.it (M.R.)

**Keywords:** aerial ecosystem, airborne microplastics, anthropogenic items, common swift, fecal sacs, terrestrial bird

## Abstract

Microplastic pollution is a pervasive global issue affecting various ecosystems. Despite the escalating production and well-documented contamination in both aquatic and terrestrial environments, the research focused on airborne microplastics and their interaction with terrestrial birds remains limited. In this study, we collected fecal sacs from Common swifts (*Apus apus*) to investigate their diet and to evaluate the potential ingestion of microplastics by both adults and nestlings. The diet was mainly composed of Hymenoptera and Coleoptera and did not differ among sexes and age classes. The 33% of nestlings’ and 52% of adults’ fecal sacs contained anthropogenic items, the totality of which was in the shape form of fibers. The 19.4% of the anthropogenic items were chemically characterized as microplastics, either polyethylene terephthalate (PET; two microfibers) or cellophane (four microfibers). Airborne anthropogenic items, including microplastic, might be passively ingested during the Common swift aerial feeding. In addition, our findings suggest that these ingested microparticles have the potential to be transferred to the offspring through food. While further research is essential to elucidate the pathways of microplastic ingestion, our results reinforce the evidence of the transfer of anthropogenic items from the atmosphere to the biota.

## 1. Introduction

At the global scale, plastic production has surged from 1.5 million tons in 1950 [1] up to 400.3 million metric tons in 2022 [2], approximately 62% of which constitute waste that has either accumulated in landfills or dispersed into the environment [3]. Once in the environment, plastic waste undergoes different degradation processes, resulting in the fragmentation of plastic debris into items of variable sizes, shapes, and polymer compositions [4]. Since the early 2000s, researchers have focused their attention on studying microplastics, defined as any synthetic solid particle or polymeric matrix insoluble in water, with regular or irregular shape and size ranging from 1 μm to 5 mm, of either primary or secondary origin [5]. Nowadays, microplastics stand as major contributors to global pollution, impacting various ecosystems, including marine, freshwater, and terrestrial environments, and extending to remote areas such as deep-sea sediments [6], arctic ecosystems [7], and mountain glaciers [8,9,10]. All these ecosystems’ microplastics, thanks to their small dimensions, are readily taken up by a wide range of animal species spanning different trophic levels and employing various feeding strategies [11].

Since the first evidence of microplastics presence in the Sargasso Sea [12], the scientific community has focused on evaluating their presence in marine and freshwater environments. Conversely, significantly less attention has been dedicated to investigating the distribution and biological impacts of microplastics on terrestrial environments, despite evidence suggesting that microplastic contamination on land may be 4–23 times greater than in the ocean [13]. This research gap hinders our understanding of the sources, prevalence, and fate of microplastics in these environments [14,15,16] that are considered the primary reservoirs for microplastics [17,18,19]. Microplastics may enter the terrestrial environments through different mechanisms such as agricultural film breakdown, sewage sludge application, organic manure usage, water irrigation, atmospheric deposition, surface runoff, as well as from the breakdown of larger plastic litter in the environment [20]. To date, the bulk of the research in the terrestrial environment has focused on soil pollution, with documented evidence of microplastics’ presence and effects across a diverse array of animal orders, including opisthopora, nematodes, collembola, tubificida, isopoda, oribatida, stylommatophora, and earthworms [21,22]. The microplastics in terrestrial environments can be carried upward by the wind, where they accumulate above the ground and undergo resuspension. These suspended particles stem from diverse sources, including domestic, industrial, and agricultural activities [23]. Conversely, research on microplastic contamination in the atmosphere remains limited, and there are currently no standardized methods for sampling MPs [24,25]. Most airborne microplastics identified globally consist predominantly of fibers from such materials as polypropylene, polyethylene, polystyrene, polyethylene terephthalate, and polyester. Their concentrations range from 4 to 11,130 particles/m^2^, with sizes from 5 to 9554 μm [26]. To investigate the presence of MPs in the atmosphere, active and passive sampling methods are the primary approaches currently used [25]. Active sampling involves using pumping sampler systems to draw a controlled amount of air over a certain period of time through a filter, allowing for the calculation of particulate matter quantity or concentration. This method is fast, accurate, and suitable across various locations. In contrast, passive sampling is simpler and cheaper, allowing for the collection of atmospheric deposition in containers or through dustfall collection by means of adhesive surfaces, suitable for long-term outdoor use without power. Despite its advantages, passive sampling has drawbacks, such as contamination by vegetation or insects, vulnerability to vandalism, or chemical contamination from adhesives. Furthermore, passive sampling may selectively capture larger airborne particles, potentially underestimating the presence of smaller microplastics suspended in the air for extended periods [27]. Another potential approach could entail employing model species to monitor airborne microplastic contamination, mirroring established practices in a water environment [28], where several invertebrate and vertebrate species have been proposed as sentinel organisms and/or bioindicators of microplastic pollution [29].

Birds are an ideal biological model for assessing airborne microplastics. Indeed, they are already widely utilized as biological indicators for evaluating air, water, and soil contamination by various pollutants, such as pesticides, heavy metals, and polychlorinated biphenyls [30,31]. Their well-documented taxonomy and biology, ease of detection, monitoring, sampling, global distribution mirroring that of many other wildlife groups, specialized habitat requirements, economic importance, and general support for their conservation efforts make birds excellent biological indicators [32]. In aquatic ecosystems, numerous studies have underscored the pervasive occurrence of microplastics within the digestive systems of seabirds, which can be directly ingested from the aquatic medium or can enter food chains through trophic transfer [33]. Conversely, less research has addressed the ingestion of micro- and mesoplastics by terrestrial birds [34,35,36,37] despite the likelihood of anthropogenic particle ingestion among these species.

The Common swift (*Apus apus*) is a promising biological indicator of airborne microplastic contamination in urban and peri-urban environments due to several distinctive traits. Specifically, this long-lived species, with a lifespan of up to 21 years [38], forms breeding colonies across Europe and spends most of its life, including mating and sleeping, on the wings. Its diet consists of airborne arthropods, including Diptera, Coleoptera, Hemiptera, and Hymenoptera. Those are captured within an altitude range that spans from ground level to approximately 100 m above ground level [39]. Furthermore, previous investigations have highlighted the suitability of this bird as a sentinel species for monitoring the levels of diverse chlorinated contaminants, including polychlorinated biphenyls (PCBs), polychlorodibenzop-dioxins (PCDDs), polychlorodibenzofurans (PCDFs), homologs of dichloro-diphenyl-trichloroethane (DDTs), and hexachlorobenzene (HCB) [40,41,42].

The peculiar feeding strategy of the Common swift, characterized by its mouth opening wide to ingest insects from the air column, may result in an intake of microplastics suspended in the air column during feeding, potentially accumulating in its digestive tract. However, the lack of information on microplastic intake by Common swifts in the literature limits our ability to determine its reliability as a biological indicator for assessing airborne microplastics. In this work, we examined the diet of common swift adults and nestlings through the analyses of fecal sacs. In addition, we also aimed at determining the ingestion of airborne microplastics by adult birds and the potential transfer of these contaminants to their offspring.

## 2. Materials and Methods

In spring 2021, we captured Common swifts in a colony breeding within a historical ‘swift tower’ in the municipality of Jerago con Orago (45°71′ N, 8°61′ E; Varese, Northern Italy). The tower hosts 105 artificial cavities, easily accessed from inside the tower, used by Common swifts for nesting. Breeding adults and nestlings were captured during the night in their nests. As the Common swifts lack sexual dimorphism, a blood sample for molecular sexing was collected from both adults and nestlings by puncturing the brachial vein. Blood was collected in heparinized capillary tubes, kept fresh in the field, and frozen at −20 °C within a few hours of collection. Sex was determined through polymerase chain reaction (PCR) amplification of the sex-specific avian CHD-1 gene [43].

Voluntarily excreted fecal sacs from both adults and nestlings (*n* = 31; 6 from adults and 25 from nestlings) were collected into plastic Petri dishes lined with aluminum foil and subsequently stored at −20 °C until laboratory analyses to isolate and characterize the diet and the microplastic contamination. Throughout the field sampling process, a field blank was obtained by placing a Petri dish lined with aluminum foil near the operator. All the animals were immediately released back into their nests after sample collection.

The research conducted in this study complied with the animal welfare laws of Italy (Regione Lombardia, Decreto N. 1043/2021; Provincia di Varese, Identificativo atto n. 537/2019). All applicable guidelines for the care and use of animals were followed.

### 2.1. Isolation of Microparticles

To avoid laboratory contamination, all the glassware, stainless forceps, and pins used were washed with acetone, rinsed with ultrapure water (filtered on cellulose filters; StonyLab, pore size 1 μm; Ø = 47 mm), and then wrapped in tinfoil until analyses. Fecal samples were processed following an adjusted protocol previously developed for the analysis of microplastic presence in Kingfisher (*Alcedo atthis*) pellets [44]. Considering that fecal sacs are formed by a closed sac surrounded by a layer of mucus, to avoid an overestimation of ingested microplastics due to external contamination, any item adhering to the external surface of the sac was removed using stainless pins.

To check for the ingestion of microplastics, each fecal sample was opened with stainless pins, and the content was inspected using a Leica EZ4 W stereomicroscope (Leica Microsystems, Deerfield, IL, USA) to identify anthropogenic items following a well-known protocol [8,45]. In detail, anthropogenic items were grouped into four main categories according to their shape (i.e., fragments, fibers, foams, and films), and a Munsell chart was used to specifically assign a color to each item. For the Munsell chart, eight broad color designations (i.e., black, blue–purple, white–transparent, grey–silver, orange–brown, green, red–pink, and yellow) were used to which items were assigned the following color gradients to determine a light or dark tone classification [46].

The inspection and isolation limit of the anthropogenic items was 20 µm in size, following a method used for bird regurgitates [45]. 

Any anthropogenic item identified from each fecal sac was hand-picked and transferred to a cellulose membrane filter (StonyLab, New York, NY, USA, pore size 1 μm; Ø = 47 mm) and placed into a glass Petri dish using stainless pins. After the transfer to the cellulose membrane filter (StonyLab, pore size 1 μm; Ø = 47 mm) to clean each anthropogenic item from the potentially associated organic matter residuals, a chemical digestion was carried out. Each item was digested overnight with 500 µL of a pre-filtered (on cellulose filters, StonyLab, pore size 1 μm; Ø = 47 mm) 15% hydrogen peroxide solution. Subsequently, each cleaned anthropogenic item was manually transferred to an Anodisc membrane filter (Whatman^®^, Maidstone, UK; Ø = 13 mm, pore size = 0.2 μm) with stainless pins. Each filter was then photographed to measure the maximum length and to confirm the color and shape of each anthropogenic item. The measurements were carried out using the freeware software ImageJ Fiji (Version 1.54i) [47]. Besides the field blank, to check for potential aerial contamination from the laboratory environment, an additional blank was performed. In detail, following Winkler et al. [44], during all the laboratory activities, a cellulose filter was placed into an open glass Petri dish next to the equipment. Both the field blank and the laboratory blank were processed as described above to check for external contamination due to anthropogenic items.

**Figure 1:**
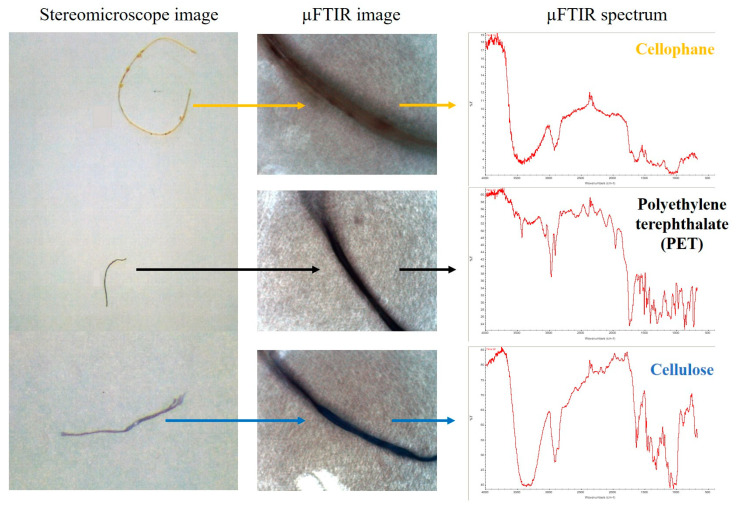
Example of the stereomicroscope image, the µFTIR image, and the µFTIR spectrum obtained through the µFTIR analyses for each anthropogenic item.

To disentangle microplastics among the isolated anthropogenic items, the polymer characterization of each item was carried out through microscopy coupled with Fourier Transformed Infrared Spectroscopy (µ-FTIR) using a Nicolet iN10 MX Infrared Imaging Microscope (Thermo Scientific, Waltham, MA, USA). The analysis was performed in reflection mode by using a wavenumber range of 4000–650 cm^−1^. OMNIC™ Picta 1.9 software (Thermo Scientific, Waltham, MA, USA) controlled the instrument. For each single spectrum, a total of 256 scans were acquired, with a spectral resolution of 4 cm^−1^. Different libraries were used for polymer identification, such as HR Aldrich Polymers, HR Coatings Technology, HR Hummel Polymer and Additives, HR Industrial Coatings, HR Polymer Additives and Plasticizers, HR Rubber Compounding Materials, HR Spectra Polymers and Plasticizers, Hummel Polymer sample Library, and Polymer Laminate Films. For each anthropogenic item, a µFTIR image and a µFTIR spectrum of the identified polymer were obtained (Figure 1).

### 2.2. Diet Determination

After the isolation of anthropogenic items, fecal sacs were spread on Petri dishes filled with ethanol 70% and inspected under a Leica EZ4 W stereomicroscope to extract each insect body part (e.g., heads, thorax, legs, elitrae, abdomens, etc.) or any other cuticle fragment that could be recognized as belonging to a given body part. Similarly to all bird species, Common swifts are not able to digest chitin, hence allowing for the use of most—if not all—insect body fragments found in the feces for taxonomic identification of prey [48,49]. Insect prey were identified at the order and family level using taxonomic keys [50,51,52]. The number of prey items from each taxon was established by applying the rule of summation of different chitin parts to the level of one individual [49].

### 2.3. Statistical Analyses

The homogeneity of the multivariate variance in the diet composition between sexes and age classes was evaluated using the betadisper function in the R package Vegan [53,54]. Differences in the diet of males and females, as well as between adults and juveniles, were further investigated by a redundancy analysis (RDA). As we were more interested in the relative composition of the diet compared to the absolute number of insect prey, all the multivariate analyses were performed using the Hellinger distance [55,56]. Further, the presence and the number of anthropogenic items in the fecal sacs were modeled by a generalized linear model assuming, respectively, a binomial and a Poisson distribution of the data according to the individual’s sex, age class, and the number of insects found in the fecal sac. All the analyses were performed in R 3.6.2 [57].

## 3. Results and Discussion

**Figure 2:**
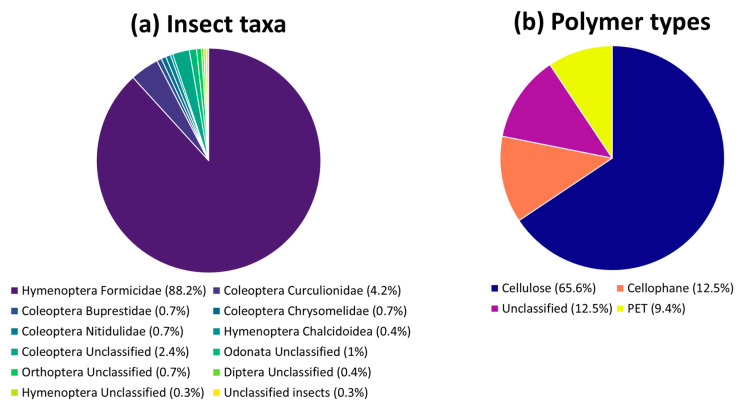
Proportion of insect taxa (**a**) and polymer types (**b**) identified in the fecal sacs of adult and nestling Common swifts of either sex.

A total of six adults and 25 nestlings were sampled for this work. Molecular sexing identified 3 males and 3 females among the adults and 10 males and 13 females among the nestlings; due to the unavailability of blood samples, molecular sexing could not be performed for two nestlings. Overall, 293 prey items were found, 292 of which were classified at the order level and 278 at the family level. The diet was mainly composed of Hymenoptera (259 specimens) and Coleoptera (25 specimens), mainly represented by families of Formicidae (257 specimens) and Curculionidae (12 specimens), respectively (Figure 2). The observed relative proportion of different insect taxa found in the fecal sacs aligns with the results of previous studies conducted in different Common swift populations across Europe, whereby Coleoptera, Hymenoptera, and Homoptera were the most represented taxa [39,58,59,60,61]. The multivariate variance of the diet was homogeneous among sexes and age classes (F_4,26_ = 0.63, *p* = 0.63), and the RDA indicated no significant differences in diet composition among the groups (F_4,26_ = 0.81, *p* = 0.61). These findings may suggest the lack of sex-related differences in both the ability of adults to exploit the food sources and their foraging behaviors. However, given our small sample size, especially concerning adult birds, all our results should be interpreted cautiously. Likewise, the homogeneity in diet among nestlings may suggest that parents feed them regardless of their sex. Finally, adults seem to feed nestlings with the same prey they catch for themselves.

Anthropogenic items were detected in 15 out of the 31 (48%) fecal sacs collected, for a total of 31 items. All the isolated items were fibers. Neither the sex nor the age class of the individuals showed any association with the presence or the number of fibers found in the fecal sacs (Table 1). The absence of age-related microplastic (MP) ingestion in this study might suggest that fibers do not accumulate in the gastrointestinal tract of Common swifts. Furthermore, studies examining age differences in microplastic ingestion have yielded inconsistent results, with some showing no differences among age classes [62,63], while others observed higher levels of ingested microplastics in juvenile [64,65] or adult birds [66]. Similarly, the absence of differences between males and females in plastic ingestion aligns with previous research on other bird species, such as the Canadian Arctic northern fulmar (*Fulmarus glacialis*) [67] or Cassin’s Auklet (*Ptychoramphus aleuticus*) [68].

The further polymeric characterization highlighted that only 5 out of 31 (16%; one adult and four nestlings) fecal sacs contained microplastics, all in the shape of fibers (hereafter called microfibers). No microfibers were found for the laboratory blank, while for the field blank, a microfiber was found (i.e., polystyrene). However, as the fecal sac can be considered as a closed sac surrounded by a layer of mucus, this single microfiber found in the field blank did not overestimate the abundance of microfibers ingested by Common swift. Regarding the polymeric characterization, two out of the 31 fibers (6%) were identified as polyethylene terephthalate (PET), 4 as cellophane (13%), 21 as cellulose (68%) while 4 remained as unclassified (13%) (Figure 2). In detail, regarding the microfiber colors, the majority were transparent (67%), followed by black (17%) and white (17%). The microfibers isolated from the nestling fecal sacs presented a mean dimension (±standard deviation) of 2.16 ± 1.55 mm (range 4.04–0.95 mm), while the fecal sac of the adult contained a single microfiber, with a length of 3.33 mm.

The relative abundance of cellophane and PET aligns with the few studies investigating the presence of anthropogenic microparticles in terrestrial birds. For instance, Carlin and co-workers [34] quantified the abundance of microplastic contamination in the gastrointestinal tract of eight bird of prey species. In detail, the number of microplastics found was highly variable, ranging from 0.16 to 20.8 fiber and from 0 to 3.23 fragments per species, while regarding the polymeric composition, they highlighted the presence of different polymers such as cellulose, PET, polyethylene, polypropylene, styrene-ethylene-butylene, and polyamide. Moreover, the polymers found in Common swift fecal sacs align with another study carried out by Hoand and Mitten on six passerine bird species, which highlighted polyethylene, PET, nylon, and polyvinyl fibers as the dominant polymer type found in the gastrointestinal tract of the adult individuals analyzed, with a length ranging from 0.01 to 20 mm [35]. Analogously, Sherlock and co-workers carried out a study on Tree swallow (*Tachycineta bicolor*) nestlings, highlighting fibers as the dominant microplastic shapes found both in the gastrointestinal tracts and in the feces of birds, mainly composed of polyurethane, polypropylene, polyethylene, polyethyleneimine, and polyethylene terephthalate with a dimension ≤ 6 mm [36]. In addition, besides the presence of microfibers in the fecal sacs of adults, the finding of microfibers in nestlings’ fecal sacs, which in this stage of life strongly depend on the parental supplied diet, highlighted the potential transfer of airborne microplastics from parents to the offspring. This result agrees with a previous study by Carey in 2011 [69], who highlighted the transfer from parents to fledging of synthetic material in Australian colonies of the seabird Short-tailed shearwaters (*Ardenna tenuirostris*). Moreover, Hoang and Mitten in 2022 [35] suggested that the presence of microplastics in the digestive tracts of Tree swallow nestlings could be derived from a potential transfer from the parentally supplied diet. Overall, besides the polymeric nature (i.e., plastic or natural) of the anthropogenic item found, the prevalence of fibers in our samples aligns with previous research, emphasizing fibers as the predominant shapes detected in the atmosphere [26], as well as the dominant types in terrestrial birds [62]. Originating mainly from textile materials, these microfibers become airborne with winds and settle in diverse ecosystems worldwide [70]. However, since our investigation exclusively focused on microparticles recovered from birds’ feces, we cannot rule out the possibility that the Common swifts also ingest microparticles with other shapes. For instance, this could be attributed to limitations in our microplastics isolation method, which excludes particles smaller than 20 µm from analyses due to the magnification of the instrument used for the visual inspection. In addition, while fibers could be more easily excreted, other microparticle shapes, such as fragments, may not undergo complete excretion, leading to potential accumulation. This phenomenon was observed in a recent study on Tree swallows by Sherlock and colleagues [36]. In their research, although only fibers were recovered in the feces, the gastrointestinal tracts of the analyzed nestlings also contained microplastic pellets and fragments. Regrettably, detecting the presence of any additional microplastics within the bird’s body (including lungs, air sacs, or any other part of the gastrointestinal tract) requires sacrificing the animal, a procedure we chose not to conduct for this study.

Finally, we hypothesized that the Common swifts might ingest microplastics through two pathways: either passively while feeding or by ingesting contaminated insects. The findings of this study align with the first hypothesis, suggesting that these birds passively ingest airborne microplastics while feeding with their mouth wide open. The second hypothesis is not supported by our data, following the lack of association between the presence or number of microfibers and the number of insects in their fecal sacs (Table 1), coupled with the fact that the identified microfibers have a dimension that prevents the ingestion by the insects constituting the Common swifts’ diet [71]. However, despite our results appearing to be in agreement with the first hypothesized ingestion pathway, supporting the idea that the Common swifts’ foraging strategy contributes to the ingestion of microfibers, a larger sample size should be necessary to effectively disentangle these two—potentially complementary—hypotheses. In addition, future studies should be conducted to perform a comparative analysis among various Common swift colonies to validate the consistency of our results on a larger spatial scale.

In conclusion, the findings of this study shed light on the dietary habits and microplastic ingestion patterns of Common swifts, offering insights into their foraging behavior and exposure to anthropogenic items. The identification of various plastic polymers, including polyethylene terephthalate and cellophane, underscores the widespread airborne contamination by synthetic materials. Notably, the presence of microfibers in nestling fecal sacs suggests a transfer of airborne microplastics from parents to offspring, highlighting the need for further investigation into the mechanisms of exposure. Therefore, additional studies are needed to better address the plastics’ ingestion pathways, their accumulation within the avian digestive system, and even the potential negative effects on the organisms. Finally, this study reinforces the evidence of the transfer of anthropogenic items from the atmosphere to the biota, highlighting the importance of ongoing efforts to monitor the microplastic pollution in the ecosystems, recognizing the vulnerability of avian species such as the Common swift to environmental contamination.

## Figures and Tables

**Table 1:** Generalized linear model of the presence and number of microfibers found in the fecal sacs of individuals in relation to their sex, age, and number of insects found in the feces. A binomial and Poisson distribution of the data were assumed to model the presence and the number of microfibers, respectively. Sample size was 3 adult females, 3 adult males, 13 female nestlings, 10 male nestlings. The 2 nestlings of unknown sex were excluded.

	Estimate	Standard Error	Z	*p*
**Presence/absence**				
Sex	−0.2	0.77	0.26	0.8
Age	−0.53	0.98	0.54	0.59
Number of insects	0.04	0.06	0.71	0.48
**Number**				
Sex	0.22	0.4	0.54	0.59
Age	−1.07	0.74	1.48	0.15
Number of insects	0.03	0.03	1.28	0.2

Bold represents the response variable, while sex, age and number of insects are the predictors.

## Data Availability

The data are available from the corresponding author upon request.
